# Supplementary material for: A Global Screen for Assembly State Changes of the Mitotic Proteome by SEC-SWATH-MS
Source: Cell Syst. 2020 Feb 26;10(2):133–155.e6. doi: 10.1016/j.cels.2020.01.001 (PMC7042714; doi:10.1016/j.cels.2020.01.001)

O95376 | ARI2_HUMAN | ARIH2 ARI2 TRIAD1 HT005
Monomer MW [kDa]: 57.819 Monomer expected elution fraction: 45

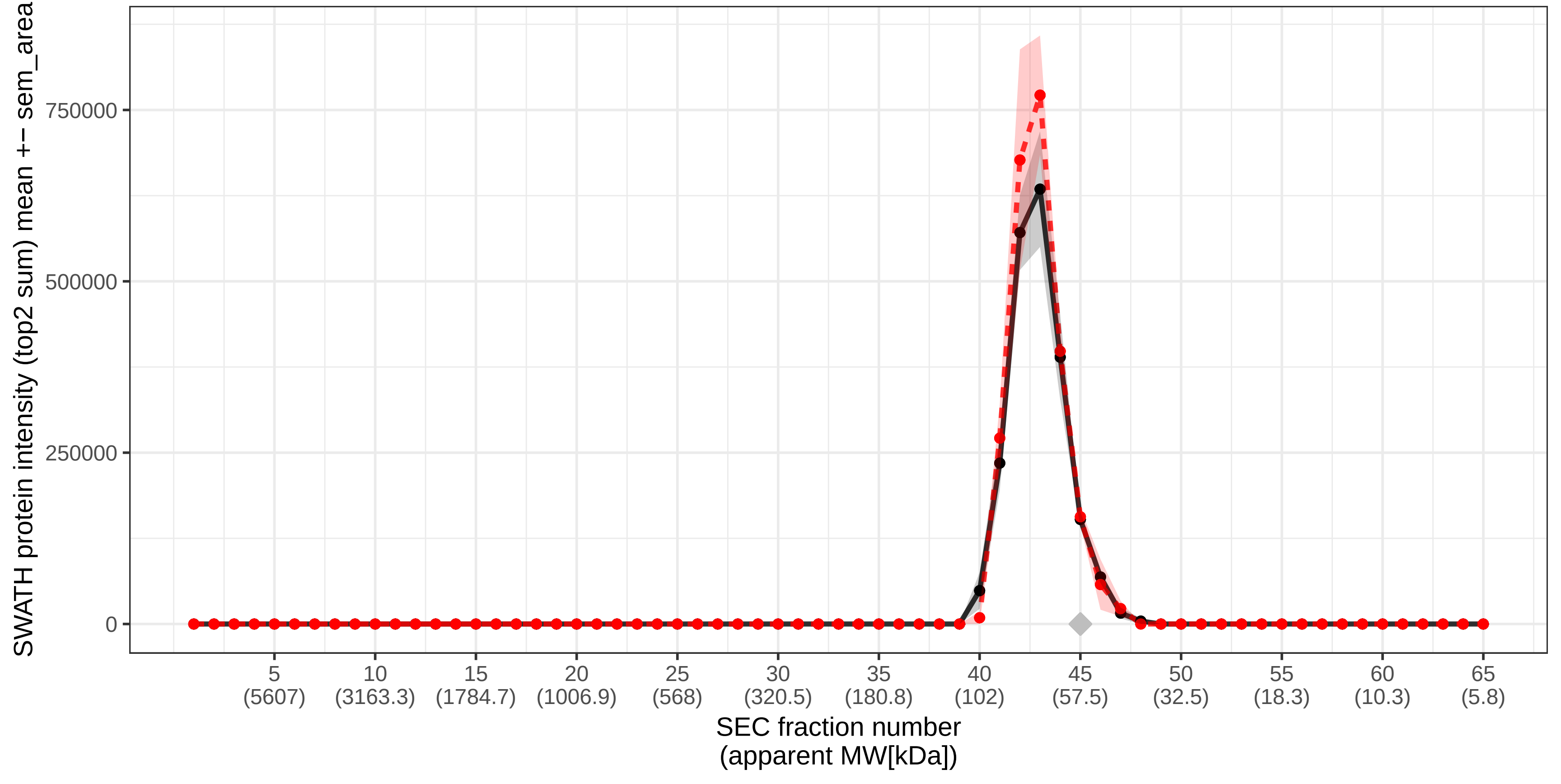

Supplement: Data S1. SEC-SWATH-MS Protein Chromatograms, Related to Figure 1 [file mmc6.zip › SECchrom_O95376_ARI2_HUMAN_ARIH2_ARI2_TRIAD1_HT005.pdf]
